# Mix Frame Visual Servo Control Framework for Autonomous Assistive Robotic Arms

**DOI:** 10.3390/s22020642

**Published:** 2022-01-14

**Authors:** Zubair Arif, Yili Fu

**Affiliations:** State Key Laboratory of Robotics and Systems, Harbin Institute of Technology, Harbin 150001, China; zub@hit.edu.cn or

**Keywords:** visual servo control, assistive robotic arms, IBVS, autonomous robots, mix frame Jacobian matrix

## Abstract

Assistive robotic arms (ARAs) that provide care to the elderly and people with disabilities, are a significant part of Human-Robot Interaction (HRI). Presently available ARAs provide non-intuitive interfaces such as joysticks for control and thus, lacks the autonomy to perform daily activities. This study proposes that, for inducing autonomous behavior in ARAs, visual sensors integration is vital, and visual servoing in the direct Cartesian control mode is the preferred method. Generally, ARAs are designed in a configuration where its end-effector’s position is defined in the fixed base frame while orientation is expressed in the end-effector frame. We denoted this configuration as ‘mixed frame robotic arms’. Consequently, conventional visual servo controllers which operate in a single frame of reference are incompatible with mixed frame ARAs. Therefore, we propose a mixed-frame visual servo control framework for ARAs. Moreover, we enlightened the task space kinematics of a mixed frame ARAs, which led us to the development of a novel “mixed frame Jacobian matrix”. The proposed framework was validated on a mixed frame *JACO*-2 7 *DoF* ARA using an adaptive proportional derivative controller for achieving image-based visual servoing (IBVS), which showed a significant increase of 31% in the convergence rate, outperforming conventional IBVS joint controllers, especially in the outstretched arm positions and near the base frame. Our Results determine the need for the mixed frame controller for deploying visual servo control on modern ARAs, that can inherently cater to the robotic arm’s joint limits, singularities, and self-collision problems.

## 1. Introduction

The ultimate goal of science and engineering is to serve humanity and humans by creating ease in their daily lives. Robotics is an innovative engineering discipline that does so by automatically performing repetitive, laborious, and complex tasks, providing relief to humans. Recently, with the advancement in robotic technologies, the acceptance of robots in society has improved considerably, resulting in an increased human-robot interaction application [1]. Large volumes of robots are not only brought into the industry, but are also introduced in dynamic environments that were originally designed for humans, such as in homes, schools, and hospitals [2]. As these environments are dynamic in nature, such workplaces demand a high level of autonomy and dexterity, which needs to be developed in robots to perform their task autonomously [3].

Vision is the fundamental sensor that humans use to perceive, adapt, and work in dynamic environments. Henceforth, robotic vision has emerged as the vital tool for robots to perceive the environment and acquire autonomy to perform their tasks in human-centric environments in human-robot interactions (HRIs) [4].

One key area of HRI is providing care to patients, the elderly, and people with disabilities. Recently, assistive robotic arms (ARAs), a form of robotic assistive care have gained wide attention in the research community [5]. ARAs for giving care to the patients and the elderly such as in [4,5] showed that assistive robotic arms are quite effective in providing support to the people with disabilities to recover most of their autonomy.

Many companies commercially developed ARAs such as *MANUS* and *i-ARM* by Exact dynamics (Netherlands), the *WAM* arm by Barrett Technology (USA), and, the *JACO* and *MICO* series robotic arms by Kinova^®^ Robotics (Canada) [6]. A comparative study of different assistive robotic arms platforms can be found in [7]. Among these companies, Kinova^®^ robotics is the leading manufacturers of ARAs with more than 50% share in the market [8].

In this paper, we discuss the integration of visual sensors in ARAs which plays a vital role in inducing autonomous behavior. We demonstrate how visual servo control in the direct Cartesian control mode is the preferred control scheme to implement on assistive robotic arms. We also note that assistive robotic arms are kinematically different from other robotic arms that use a hybrid mixed frame configuration for their operation. Hence, conventional visual servo controllers cannot be directly deployed on ARAs. Therefore, we explored the task space kinematics of a mixed frame assistive robotic arm and developed our mixed frame visual servo control framework, which led us to the novel development of the proposed mixed frame Jacobian and the mixed frame velocity. We successfully deployed an Adaptive proportional derivative (PD) image-based visual servoing (IBVS) controller on ARAs using their embodied mix frame kinematics while safeguarding its core functionality. Our work will induce autonomous behavior in ARAs and will inherently pave the way for implementing conventional visual servo controllers on ARAs.

### Related Work: Autonomous Control Schemes for Assistive Robotic Arms

Various methods are well-documented in the literature to induce autonomous behavior in robotic arms such as picking and placing objects, assistive feeding, and sip and puff, which are frequently required activities of daily living (ADL). Several examples can be found in [4,5,9].

ARAs are useful to user with disabilities, yet face one major challenge. Most ARAs still provide dull and non-intuitive interfaces to interact with the robotic arms and thus lacks the basic autonomy required to perform (ADL). Commonly, these devices provide a joystick control of ARAs with limited preset buttons, the use of force feedback and moving the joystick can be very imprecise with users suffering from muscular weakness, consequently, it is problematic to perform ADL, given the mere amount of autonomy in control of the robotic arm. Several studies were conducted showing user dissatisfaction towards the efforts, time, and expertise needed to control these ARAs such as work carried out by Campeau et al. (2018) [10], and problems faced during joystick control are discussed by Beaudoin et al. (2018) [11]. Ka et al. (2018) [12] evaluated the user’s satisfaction using a joystick and semi-autonomous control for performing ADL.

To address this problem, several other interfaces were developed by researchers such as Poirier et al. (2019) [13] who introduced voice-controlled ARAs in an open-loop fashion. However, the voice command technique did not decrease the time to perform a task as compared with joystick control. Kuhner et al. [14] used deep learning techniques to develop brain signal computer interface (BCI) systems to control ARAs. Nevertheless, BCI methods need prior training with the user, and post and preconditioning of the signals to be used in real-time. A human user must wear detection leads which are not practical to wear all the time by the user. For the case of assistive feeding. Aronson et al., 2019 [15] added autonomy to ARAs by integrating an eye gaze tracking system.

One good way of making robotic arms autonomous is to integrate visual sensors in ARAs, resultantly, robotic arms can see and interact with their environment autonomously. Jiang et al. [16] developed a multimodal voice and, vision integrated system on a wheelchair-mounted robotic manipulator (WMRM). Law-Kam Cio et al. (2019) [17], integrated a vision sensor to ARAs using two Kinect depth cameras, one to identify the user’s face, and another for guiding the robotic arm to grab an object using the look and move method, the application was promising; however we argue that the method was computationally expensive and also an abundance of hardware was mounted on the wheelchair by using two Kinect cameras on a wheelchair, which reduces the user autonomy and mobility of the wheelchair in narrow areas around the house. Therefore, we developed our system with minimal hardware using a single camera.

For developing autonomous control of ARAs, some researchers have developed their application in the robot operating system *ROS^®^-MOVE-IT^®^* environment. Snoswell et al. (2018) [18], developed a pick and place system on Kinova^®^ MOVO dual-arm robot using a Kinect vision sensor. We argue that using a generic robot controller with a robot kinematic model defined in a separate *URDF* file, as was the case of [19], will add complexity to the system and compromise the core functionality of the assistive robotic arm such as its safety features, singularities avoidance, and self-collision avoidance behavior during operation. We propose using a dedicated manufacturer-designed kinematic controller for operating the ARAs.

Most of the applications that we discussed in this section, uses a ‘look and move’ approach in an open-loop manner [20], that is the positioning of the end-effector to a certain prescribed pose, learned through visual pose estimation methods of the desired object. Using an open-loop look and move method to position a robotic arm for probabilistic grasp may be an easier option to implement besides being computationally inexpensive. Nevertheless, it carries its shortcomings, for instance, measurements are made in an open-loop manner, hence the system becomes sensitive to uncertainties, such as a lack of positional accuracy of the robotic manipulator due to errors in the kinematic model of the robotic arm; internal errors such as wear, backlash, and other external reasons may such as, error in camera intrinsic parameters and extrinsic calibration, weak information of object 3D model, or if the object moved during the approach motion of the gripper. Hence, the reliability and accuracy of an open-loop look and move systems remain lesser than the visual servo feedback control systems [20].

Generally, an ARA is designed to provide a coarse, wide range of assistance to the user, they are not an accurate positioning device as discussed in the work by Karuppiah et al. (2018) [19]. Hence, their positional accuracy remains subpar to industrial manipulators, and thus position control cannot be solely depended upon to achieve the desired pose for grasping an object. Moreover, a major concern in ARAs and WMRM cases, is to ensure user and robotic arm’s safety [21], while preference is given for performing tasks in the direct Cartesian control approach.

Naturally, ARAs are required to operate in the Cartesian task space of its end effector to complete ADL. Hence, modern ARAs and WMRM utilize 6 degrees of freedom (DOF) or preferably 7 DOF to perform ADL in direct Cartesian control, as also discussed by Herlant (2018) [22]. Direct control is when the operator or control algorithm directly commands the position and orientation of the end effector in Cartesian space but does not explicitly specify the joint angles or velocity of each joint of the manipulator. The joint angles and joint velocities will be automatically determined by the robot controller. Hence, the direct Cartesian mode is an efficient control method for ARAs [23].

Considering these constraints, an optimal way to design an autonomous ARAs vision control system is to complement it with a closed-loop visual servo control in direct Cartesian task space, which can immunize the system against positioning errors, inherently present in ARAs.

Visual servo control can be defined as the use of visual information to control the pose of the robot end-effector relative to a target object in a closed-loop visual feedback manner. Visual-servo control schemes are primarily divided into two different methods, one that realizes visual servo in a 3D operational space also called pose-based visual servo (PBVS), and another that realizes visual servo in the 2D image space, referred to as image-based visual servoing (IBVS) [24], using a camera which may be mounted on the robotic arm as an eye-in-hand, or can be fixed in the workspace using an eye-to-hand configuration. Visual servo control literature can be found in [25], recent work, and an update on the field can be seen in [26].

During the literature review, we observed that researchers have not completely explored the potential of visual servo control to develop an autonomous ARAs system. An earlier attempt to utilize visual servo control with ARAs was performed by Kim et al. (2009) [27], in which hybrid visual servo control was deployed on an assistive robotic arm, i-ARM, this study pioneered the concept of our approach to utilize visual servo control to operate an assistive robotic arm in Cartesian control mode. However, our case differs as the assistive robotic arm used in our study is a redundant manipulator designed to operate in a mixed frame configuration using an adaptive image-based visual servo controller using 2D features only, whereas the aforementioned case of i-ARM uses a stereo camera and, 2-1/2 visual servo control requiring a mix of 2D and depth features, also it does not take into account safety features and self-collision avoidance behavior, which is implicitly ensured in our proposed framework. In a later work performed by Tsai et al. (2017) [28], a joint space visual servo control was developed in the IBVS scheme. However, in that work, research was not aimed at assistive robotic arms, rather it tested a light field camera model for image capturing, and an under-actuated 4 DOF Kinova^®^ MICO robotic arm was used, which cannot realize 6D Cartesian motion; thus, necessitating the use of a joint control scheme.

**Figure 1:**
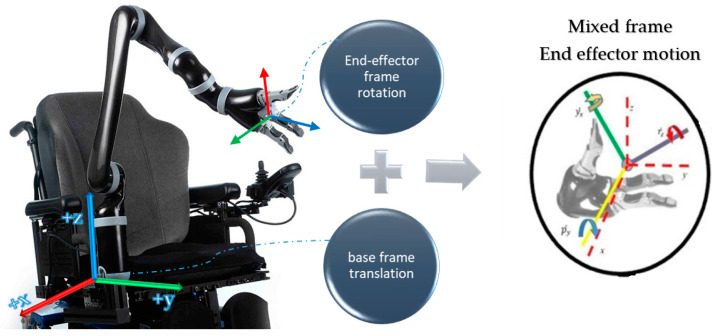
Mixed frame Configuration; a wheelchair-mounted assistive robotic arm Kinova^®^ *JACO*-2 6 DOF showing mix frame end-effector motion with translation w.r.t the fixed base frame and rotation expressed as the *R-P-Y* angles in the end-effector frame.

Mix frame robotic arms are designed to operate in an arbitrary hybrid mixed frame task space, where the end-effector position and velocity cannot be directly controlled in the end-effector frame or the base frame, rather the position of the end effector is defined in a mixed frame of reference, i.e., fixed base frame for positioning of the end effector, while the orientation is expressed as the Euler angles in the end-effector frame, as shown in Figure 1 and further discussed in Section 2. After an extensive literature review and to the best of the author’s knowledge, a formal account of the kinematics of a mixed-frame robotic arm could not be found in the literature. Therefore, the use of a mixed frame configuration in ARAs poses a major problem, which restricts the use of several mainstream control laws on mixed-frame robots. Concluding our literature review. We indicated an important problem about the absence of a visual servo control scheme for a mixed frame ARA. In pursuit of a solution, we developed a mixed frame visual servo control framework for an assistive robotic arm to devise an autonomous approach movement for picking up an object, using an adaptive gain proportional derivative image-based controller, directly controlled in the Cartesian task space. The performance is compared with the joint control scheme developed for ARAs. the experimental results showed the superiority of our proposed framework in all traits. We also developed an open-source *ViSP* library class for interfacing and controlling Kinova^®^ *JACO*-2 robots with *ViSP*^®^ [29], which is a well-known library for vision and control.

To the best of the author’s knowledge and belief, this is the first instance of work to describe a visual servo control law in a mixed frame configuration for ARAs such as the Kinova^®^ *JACO*-2. The rest of this paper is organized as follows: Section 2 formulates the task space kinematics and develops a novel mix frame Jacobian for mix frame ARAs, Section 3 develops a mixed frame image-based visual servo control framework, Section 4 describes the experimental implementation on a real 7 DOF robotic arm and discussion on the results, and Section 5 provides the conclusion of this paper.

## 2. Task Space Kinematics of a Mixed Frame Robotic Arm

### 2.1. Mix Frame Robotic Arms

Mix frame robotic arms are a category of robotic arms that uses a mixed frame configuration to represent its end effector’s pose. The end-effector Cartesian pose is given by a 6D pose vector, i.e., a combination of two vector quantities in different frames, namely the position vector of the end effector is defined in the fixed base frame, while the orientation is expressed as Euler angles in the end effector’s body frame. The use of a mixed frame configuration is beneficial because it ensures the user’s safety, self-collision avoidance behavior, and encourages intuitive interaction with the assistive robotic arms.

The mixed frame approach has various benefits as it is more perceptible for HRI engineers, due to the combination of the end-effector frame and the base frame, so a user can enjoy the intuitiveness of end-effector Euler angles for orientation and simplicity in the use of the fixed base frame for translation motion, in a single configuration. Therefore, many modern ARAs such as Kinova^®^ series *JACO, MICO, MOVO*, use this mixed frame task space control approach to achieve safe, self-collision and, singularity-free motion [30], and offer enhanced autonomous behavior [10,16,31].

In cases of ARAs and wheelchair-mounted robotic manipulators (WMRMs) [16,32], it is always feasible to design and operate robotic arms in the mixed frame. The use of mix frame methodology ensures user safety by defining stationery protective safety zones [19,33,34] around the user, sitting in a fixed position w.r.t the robotic arm base frame, while the orientation of the robotic arm is expressed in the end-effector frame. Orientation expressed in the Euler angles of the spherical wrist will aid in dealing with representational singularities and provide intuitive interaction with the ARAs.

The protection zones for a user sitting next to the robotic arm base, are associated with the fixed base frame [30,33,35]. The intuitive use of the end effector and its singularity avoidance behavior is owed to the use of the end-effector frame Euler angles and end-effector orientation in the task space. Therefore, without using the mixed frame, both of these properties cannot be simultaneously safeguarded. The mix frame Jacobian also implicitly aids in detecting and dealing with singularities as the representational singularities using the Euler angle representation are well-known prior to the instance. While using roll, pitch, and yaw angles the controller must avoid setting the middle joint of the spherical wrist, i.e., the *β* angle from obtaining 0 or pi rad (±90° for *XYZ* Euler angle case). Thus the singularity decoupling approach [36,37] can be implemented easily in the mixed frame configuration. Moreover, in mixed frame ARAs using the spherical wrist, the mixed frame Jacobian is by default partitioned in the arm and the wrist portions. Hence singularities occurring in the arm, or the wrist can be dealt with separately, and efficiently. Therefore, one can use well-established techniques for dealing with the singularities [37,38] while using a mixed-frame approach. Therefore, mixed frame direct Cartesian velocity control mode is our preferred choice of control for autonomous control of ARAs.

### 2.2. Mix Frame Task Space Kinematics

In complex and dynamic real-world applications, such as in the case of domestic environments, end-effector motion may be affected due to online adjustments, in response to sensor input to accommodate unexpected events. Thus, it is important for controlling the interaction of manipulators in a dynamic environment to utilize visual feedback in the task space.

**Figure 2:**
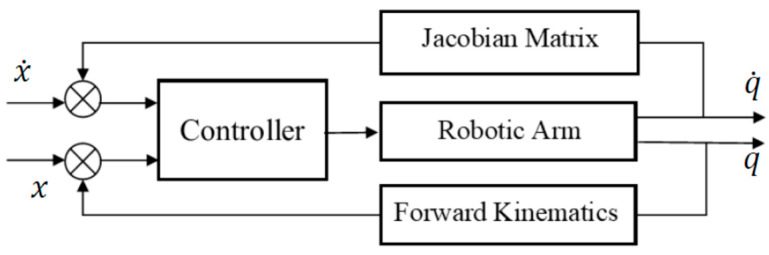
Block diagram for task space control.

The task space is a subset of the Cartesian space where the operation of the robotic arm is required, with ‘m’ DoF task such that m ≤ *n* with a maximum of 6, where ***n*** is the number of robotic arm joints. Since robotics tasks are mostly specified in the task space and demand precise control of the tool or end-effector pose and velocity, joint space control schemes are generally not suitable [39]. Therefore, operational or task space control schemes are necessary. One such scheme is shown in Figure 2, which can develop control directly based on the kinematics of the task space.

It is quite intuitive to describe the robotic arm task in terms of a manipulator end-effector pose defined by $\chi_{conv}$ which consists of the desired task, given by:

(1) $$\chi_{conv}={\left(t_{1\;}t_{2\;}t_{3}\ldots .t_{n}\right)}^{t}\;$$

where ‘$\chi_{conv}$’ represents the task space of a robotic arm in the conventional configuration, that is a set of vectors which defines a six-dimensional pose vector in 3D Cartesian space such that the first 3 terms represent the position of the end-effector and the last 3 define the minimalistic represented orientation of end effector in the task space. The representation of all translations and orientations are in one homogeneous frame of reference. For instance, consider the following $t_{conv}$ in the end-effector frame:

(2) $$t_{conv}={\left(\begin{array}{ccc} p_{x}^{e} & p_{y}^{e} & p_{z}^{e} \end{array}\;\begin{array}{ccc} \alpha_{z}^{e} & \beta_{y}^{e} & \gamma_{x}^{e} \end{array}\right)}^{t}\;$$

where ‘*p*’ defines a desired task position vector and α,β and γ represent its orientation in the task space w.r.t end-effector frame. Details on conventional task frame kinematics can be found in [36].

Alternatively, in assistive robotic arm and WMRM cases, we deal with an unusual frame configuration for the task space, which operates otherwise, using a hybrid frame, we denoted it as a ‘mixed-frame’ configuration for its task space as shown in Figure 1, which shows that the end effector uses a hybrid mixed frame for its motion, i.e., its position and translation velocity is operated in the base frame and the orientation and rotational velocity of the end effector is defined by the minimalistic represented Euler angles, w.r.t the moving end-effector body frame. Accordingly, an updated task space for mixed frame assistive robotic arms can be defined as:

(3) $$\chi_{mix}={\left(t_{m1\;}t_{m2\;}\ldots .t_{mn}\right)}^{t}$$

where:

(4) $$t_{m}={\left(\begin{array}{ccc} p_{x}^{b} & p_{y}^{b} & p_{z}^{b} \end{array}\;\begin{array}{ccc} r_{z}^{e} & p_{y}^{e} & y_{x}^{e} \end{array}\right)}^{t}$$

In Equation (4) defined task $t_{m}$ is a mixed frame representation of the task space of an assistive robotic arm, defined in the hybrid mixed frame where, p defines the position vector of end-effector w.r.t the base frame and r,p,and y represent the orientation of the end effector in roll, pitch, and yaw,(*r-p-y*) angles corresponding to the end-effector frame. Taking a time derivative of Equation (4) for obtaining velocity level kinematics, we obtain a 6 × 1 spatial twist vector $V_{e}^{m}$ which is the desired end effector’s mix frame task space velocity as proposed in this paper.

(5) $$\dot{\chi_{m}}=\left(\begin{array}{c} \nu_{e}^{b}\\ {\dot{\phi }}_{e}^{e} \end{array}\right)=V_{e}^{m}={\left(\;\begin{array}{ccc} v_{x}^{b} & v_{y}^{b} & v_{z}^{b} \end{array}\;\begin{array}{ccc} {\dot{r}}_{z}^{e} & {\dot{p}}_{y}^{e} & {\dot{y}}_{x}^{e} \end{array}\right)}^{t}$$

**Figure 3:**
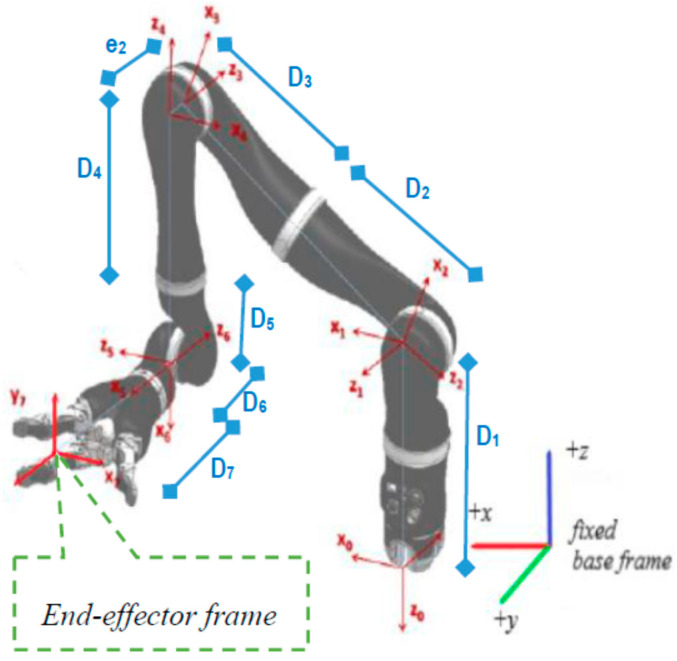
Kinova^®^ *JACO*-2 robotic arm kinematic structure and frames.

$V_{e}^{m}$ is the desired mix frame task space velocity as proposed in this paper. It has its translation velocity component expressed in the base frame, and the rotation velocity is expressed as the rate of change in Euler angles of the end-effector frame. In the case of ARAs, where most of the manipulation are performed by the end effector, usually a gripper, the task space velocities are realized in the gripper frame as shown in Figure 3. The mixed frame Kinova^®^ *JACO*-2, 7 DoF robotic arm, structural details, and *DH* parameters can be found in Table 1 and [35].

The case where a tool is mounted on the end effector such as a spoon or a camera, $V_{e}^{m}$ can be transformed to the tool frame by a fixed motion transformation matrix. This case is discussed in Section 3.

There are numerous benefits of using a mixed frame task space approach as the controller utilizes a feedback loop that minimizes task errors directly. It does not require calculating joint angles using Inverse kinematics explicitly, since the control algorithm inherits the velocity-level forward kinematics, as shown in the Figure 2. Thanks to this behavior the end effector can achieve a straight-line motion in the task space.

Conventionally, the differential kinematics relationship between the joint space velocity and the task space velocity for a conventional base frame can be written as:

(6) $$V_{e}^{b}=J_{b}\left(q\right)\dot{q}$$

where $J_{b}$ is the conventional base frame Jacobian, we used this geometric Jacobian expressed in the base frame given by:

(7) $$J_{b}\left(q\right)=\partial k/\partial q$$

where k(q) is the forward kinematics for the end-effector pose. There are numerous geometric, analytical, and numerical methods for finding this base Jacobian, which can be studied in the literature [38]. Similarly, we can re-write Equation (6) for the mix frame configuration as:

(8) $$V_{e}^{m}=J_{m}\left(q\right)\dot{q}$$

where $J_{m}\left(q\right)$ is a Jacobian matrix in the mix frame configuration. We named it the ‘Mix frame Task Space Jacobian’ of the manipulator. It is a 6 × *n* mapping matrix accounting effect of the change in velocity of the individual joint, on the end effector’s mixed frame Cartesian velocities. Analytical task space Jacobian is different as it uses homogenous frame configuration as discussed in [40]. $J_{m}$ is a Jacobian matrix expressed in the mixed frame as a compound matrix consisting of $J_{P}$, a *3*× *n* matrix relating translational velocity of the end-effector w.r.t the base frame, and $J_{o}^{e}$, a *3* × *n* matrix relating the rate of change in Euler angles of the end effector to the individual joint velocity, $J_{m}$ can be given by:

(9) $$J_{m}=\left[\;\begin{array}{c} J_{p}^{b}\\ J_{o}^{e} \end{array}\right]$$

This will eventually stack up as a 6 × *n* matrix where ‘*n*’ is the number of joints of the manipulator.

An intuitive and efficient method for computing the task space Jacobian can be found in [40]. Alternatively, if the conventional base frame Jacobian $J_{b}$ is available, the mixed frame Jacobian can be found using:

(10) $$J_{m}=J\left(\Gamma \right)\cdot J_{b}$$

where matrix J(Γ) is given by [40]:

(11) $$J\left(\Gamma \right)={\left[\begin{array}{cc} I_{3\times 3} & 0_{3\times 3}\\ 0_{3\times 3} & B{\left(\Gamma \right)}^{-1}R_{b}^{e} \end{array}\right]}_{6\times 6}$$

J(Γ) is a 6 × 6 transformation matrix [40], it is a function of task space orientation parameters of the end effector, ***I***, **0** are identity and null matrix. where Rce is a 3 × 3 rotation matrix from the base frame to the end-effector frame, Γ=(r,p,y), r = roll, p = pitch, y = yaw angles of the end-effector, and B(Γ) is defined in [40] as the mapping matrix from angular velocity to the rate of change in the Euler angles as the rotational velocity $\dot{\phi_{e}}$:

(12) $$\dot{\phi_{e}}=B{\left(\Gamma \right)}_{m}^{-1}.\omega_{e}^{e}$$

where, for the *X-Y-Z* Euler angle configuration:

$$B\left(\Gamma \right)=\left[\begin{array}{ccc} 1 & 0 & \sin p\\ 0 & \cos r & -\cos p\sin r\\ 0 & \sin r & \cos p\cos r \end{array}\right]$$

When joint space control is desired, joint velocities can be computed to implicitly control the end-effector, while ensuring the desired mixed frame task space velocities $V_{e}^{m}$ as follows.:

(13) $$\dot{q}=J_{m}^{+}.V_{e}^{m}+\left(I-J_{m}^{+}J_{m}\right)\xi$$

where $J_{m}$ is the mix frame Jacobian from Equation (10), $J_{m}^{+}$ is a Moore–Penrose (M–P) pseudoinverse of $J_{m}$, I is the identity matrix, and **ξ** is any arbitrary secondary task vector in null space for instance avoiding joint limits as in the case of [41]. Equation (13) will compute a joint velocity vector $\dot{q}$ with a minimum norm. Computing $\dot{q}$ by Equation (13) using a mix frame Jacobian will achieve $V_{m}$ in the task space.

## 3. Mixed-Frame Visual Servo Control Framework

**Figure 4:**
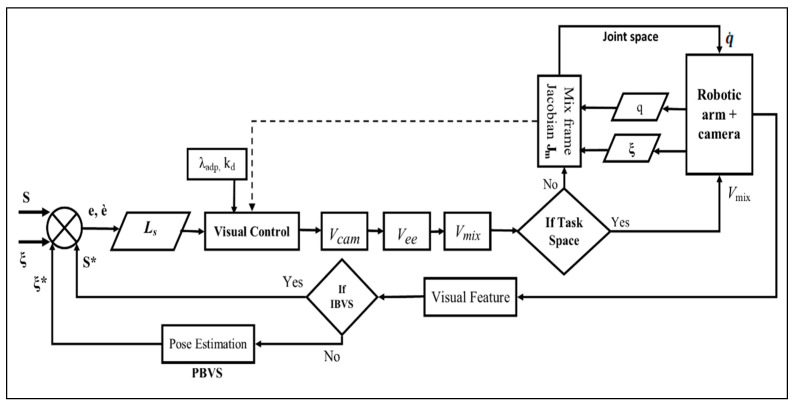
Block diagram of the proposed mix frame visual servo control framework.

In this section, we shall design a framework for deploying a visual servo control law for assistive robotic arms in a mixed frame direct Cartesian control. The proposed framework as depicted in the block diagram of Figure 4 is not constrained by the type of visual servo control scheme, that is IBVS, PBVS, or *2-1/2 D* control schemes can also be used within the framework for operating mixed frame ARAs.

Primarily, we begin with image-based visual servoing, also called 2D visual servoing. IBVS is based on the selection of a set s of the image’s visual features that need to reach the desired value $s^{*}$ in the image plane. The task is to derive the selected features and related error in the image plane to zero, the error e(t), which is typically defined by:

(14) $$e\left(t\right)=s\left[m\left(t\right),a\right]-s^{*}$$

The control schemes use the image-plane coordinate of a set of points (other features line, centroid, etc., can also be used) [25] to define the set of visual features s. Where m=(u,v) are the coordinates of the image point represented in pixel units, and the camera intrinsic parameter is given by $a=\left(u_{0},v_{0},p_{x},p_{y}\right)$ to convert pixels into meters using the *ViSP* camera projection model [24].

As a standard practice, we avoid singularities in the image interaction matrix. Let us consider a set of 4 co-planer, non-linear points P_n_ = (X, Y, Z) as image features, $s=\left(p_{1},p_{2},p_{3},p_{4}\right)$ such that each point in the image plane is given by $p_{n}=\left(x_{n},y_{n}\right)$ and $p^{*}$ is the desired image coordinates, in our case we used camera calibration parameters and geometry of target, to compute the desired image feature values of any specified goal pose [42]. The spatial velocity of the camera is denoted by, $V_{c}=\left(v_{c},\omega_{c}\right)$ with $v_{c}$ the instantaneous linear velocity of the origin of the camera frame and $\omega_{c}$ the instantaneous angular velocity of the camera frame. The relation between the rate of change in image features error and camera velocity can be given as:

(15) $$\dot{e}=L_{e}.V_{c}$$

where the interaction matrix between camera velocity and feature error $L_{s}$ is given by [24] as:

(16) $$L_{s}=\left[\begin{array}{ccc} \begin{array}{cc} -1/Z & 0\\ 0 & -1/Z \end{array} & \begin{array}{cc} x/Z & xy\\ y/Z & 1+y^{2} \end{array} & \begin{array}{cc} -\left(1+x^{2}\right) & y\\ -xy & -x \end{array} \end{array}\right]$$

In cases where visual servo control law is required to operate in joint space and angular velocities are required as input velocities to the robot controller, using a mix frame task Jacobian approach, the articular velocity $\dot{q}$ of the robotic arm can be calculated by [36]:

(17) $$\dot{q}=-\lambda (X_{e}^{c}{\hat{L_{s}J_{e})}}^{+}.e+P_{\lambda }g$$

where $J_{e}$ is the end-effector frame Jacobian,$\;\hat{L_{s}}$ is the approximation of the interaction matrix which can be formed using constant the goal frame or varying current frame Jacobian approximation technique,$\;X_{e}^{c}$ is a 6 × n motion transformation matrix to convert from end-effector frame to the camera frame, e is the task feature error and the projector operator $P_{\lambda }g$ deals with secondary tasks such as avoiding joint limit, singularities, and self-collision avoidance [41]. Now for the mixed frame configuration Equation (17) can be rearranged as Equation (18), where $J_{m}$ is the mixed frame Jacobian from Equation (10) and $X_{m}^{c}$ is the transformation matrix from the mixed frame to the camera frame.

(18) $$\dot{q}=-\lambda (X_{m}^{c}{\hat{L_{s}J_{m})}}^{+}.e+P_{\lambda }g$$

However, in this work, we are interested in producing a visual servo control law that produces Cartesian task space velocities in the mixed frame. Therefore, we will be considering the control law in direct Cartesian velocity control and deal with proportional error decay and moving target compensation given by [36] as:

(19) $$V_{c}=-\lambda \hat{L_{s}^{+}}e-\hat{L_{s}^{+}}\frac{d\hat{e}}{dt}$$

where in Equation (19), λ is a real-valued positive decay factor as a constant gain, $\hat{L_{s}^{+}}$ is the pseudo-inverse of the approximation of the interaction matrix and e is the image feature error to regulate, where the term $\hat{\frac{\partial e}{\partial t}}$ expresses the time variation in error approximation due to the target motion for the case of a non-static target, here $\hat{L_{s}^{+}}$ ∈ R ^(6×k)^ is chosen as the Moore–Penrose pseudo-inverse of the approximation of $L_{s}$ such that $\hat{L_{s}^{+}}={\left(L_{s}^{T}L_{s}\right)}^{-1}L_{s}^{T}$, when $L_{s}$ is of full rank, i.e., feature points k ≥ 6 and $\det L_{s}\neq 0$, this ensures the Cartesian space velocity to be minimal in the task space. There are several choices available for estimating the depth ‘Z’ of the image point for constructing the $\hat{L_{s}^{+}}$. We used the current visual feature depth approach for approximating the interaction matrix as described in [24].

### 3.1. Adaptive Proportional Derivative IBVS Controller

A better control law can be adopted for the system in consideration, as only a simple proportional controller is not able to minimize error with a high convergence rate to the desired low norm error. The proportional controller has a known problem of residual steady-state error or local minima convergence during visual servoing, also it has a large overshoot problem for high proportional gain, for achieving a faster rate of convergence in the time domain. Therefore, combining the proportional law with a derivative controller, which can work on the rate of change in error function would certainly help in decreasing the overshoots and decreasing the settling time [43,44].

Another problem that we encountered during the execution of IBVS control on the Kinova^®^ *JACO*-2 robotic arm in direct Cartesian control mode was, slow or no movement near the task convergence zone, as it does not move for minimal Cartesian velocities computed by the control law near convergence zone.

Therefore, the control law in Equation (19) can be further improved by taking into account an adaptive gain visual servo controller. In practice, we want our adaptive controller to produce nominal camera velocities when the task error is significant. On the other hand, a large gain value is needed when our feature error is in the convergence zone since camera velocities produced by a constant gain controller were not sufficient to converge the task as it may be stuck in local minima and not have fast decay response. We needed a larger value of gain near the convergence zone to produce higher camera velocities to achieve the task convergence rapidly.

While designing an adaptive gain for the controller, we shall rely on the task’s inherent infinity norm. Considering a range of gain values tuned between two different peak gain values, one for the case when feature error is very large, near infinity, where already camera velocities are higher. Another one for the case, where the image feature error is near zero in the convergence zone and the camera velocity is very small. For ensuring this behavior, we can replace the constant gain term with an adaptive gain as developed in [45]:

(20) $$\lambda_{adp}\left(∥e∥\right)=\left(\lambda_{0}-\lambda_{\infty }\right)e^{-\frac{\lambda_{0}^{\prime }}{\lambda_{0}-\lambda_{\infty }}∥e∥}+\lambda_{\infty }$$

where,

${∥e∥}_{\infty }$ is the infinity norm of the feature error vector.

$\lambda_{0}=\lambda \left(0\right)$ is the gain in 0 for very small values of ∥e∥.

$\lambda_{\infty }=\lambda_{∥e∥\to \infty }\lambda \left(∥e∥\right)$, is the gain to infinity, that is for very high values of ∥e∥.

$\lambda_{0}^{\prime }$ is the slope of λ at ∥e∥=0.

IBVS velocity controller in Equation (19) can be updated using a proportional derivative adaptive gain controller to move the current feature points towards desired image points by moving the end-effector mounted camera with a Cartesian task space velocity given by:

(21) $$V_{c}=-\lambda_{adp}\hat{L_{s}^{+}}\left(e+\frac{k_{d}}{\lambda_{adp}}\dot{e}\right)-\hat{L_{s}^{+}}\frac{d\hat{e}}{dt}$$

where $\lambda_{adp}$ is the adaptive proportional gain, $k_{d}$ is the derivative control gain and both are symmetric positive gain matrix of appropriate dimensions, $\dot{e}$ is the change in feature error due to eye-in-hand camera motion at each iteration of the control cycle.

The problem of residual error can also be improved using an adaptive gain proportional derivative controller, where the gain values were adapted from the visual feature error norm and the gain was continuously tuned to achieve a low constant zero error. The Euclidean error norm was calculated from the visual features error vector, where a Euclidean error norm of 0.00005 m in the image plane was used as a convergence threshold for successful IBVS task achievement.

The usage of an adaptive gain proportional derivative controller Equation (21) rather than a constant gain proportional controller for visual servo control law in Equation (19) will result in a better performance of the controller, for avoiding local minima and, a reduction in convergence time is observed in the experimental results in Section 4.

### 3.2. Mixed Frame Visual Servoing

**Figure 5:**
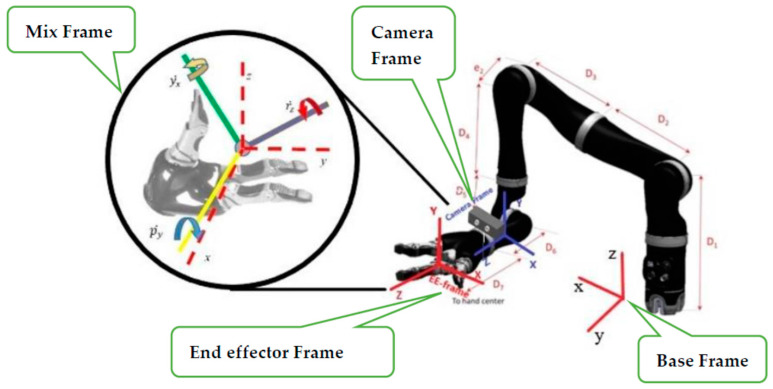
Mix frame configuration for Kinova^®^ *JACO*-2 for an eye-in-hand visual servo control.

The image-based visual servo control law derived in Equation (21) will ensure an exponential decoupled decrease in the image feature error by producing a minimum norm Cartesian velocity $V_{c}$, in the camera frame for the eye-in-hand manipulator which needs to be implemented in the mixed frame configuration. For most practical applications, the camera is usually mounted on an end effector with a constant transformation as shown in Figure 5, which can be obtained through camera extrinsic calibration [29].

Therefore, camera velocities should be transformed to end-effector velocities $V_{e}$ by using a constant spatial motion transformation matrix.

(22) $$V_{e}^{e}=\left(\begin{array}{c} v_{e}^{e}\\ \omega_{e}^{e} \end{array}\right)=X_{c}^{e}.^{c}V_{c}$$

where:

Vee=(veeωee), Xce=[Rce[tce]×Rce03×3Rce]6×6

$X_{c}^{e}$ is a 6 × 6 spatial motion transformation matrix as a function of camera pose w.r.t end effector. Where Rce is a 3 × 3 rotation matrix from the camera frame to the end-effector frame and ${\left[t_{c}^{e}\right]}_{\times }$ is the skew-symmetric matrix of the camera frame translation vector [25]. This motion transformation matrix converts the camera frame velocity to an equivalent spatial velocity $V_{e}$ which can be applied in the end-effector frame to ensure camera motion as computed by visual servo control law.

**Figure 6:**
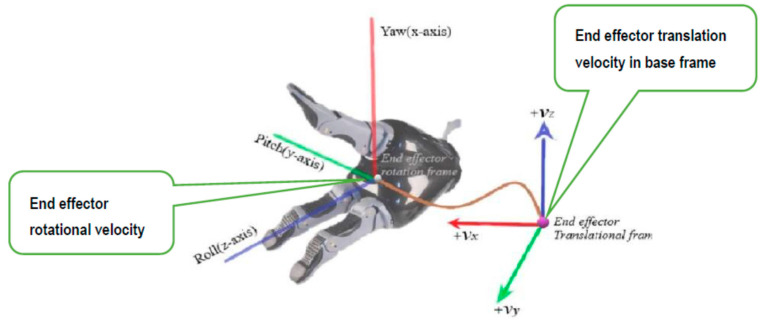
The velocity of the end effector in the Mixed frame configurations.

The velocity vector $V_{e}^{e}$ obtained in Equation (22) provides the velocity of the end-effector frame w.r.t end-effector body frame. This is the end-effector velocity conventionally used as input to the controller for materializing image-based control in industrial robotic arms. However, for an ARAs operating in a mixed frame, this end-effector velocity was not applicable and should be converted to mix frame velocity $V_{e}^{m}$, i.e., translation velocity w.r.t fixed base frame and rotation velocity in the end-effector body frame as the rate of change in roll, pitch, and yaw angles as shown in Figure 6.

For converting this end-effector velocity into the desired mix frame velocity, first, we can convert end-effector velocity to the base frame using a rotation matrix Reb which can be obtained by the pose of the end-effector w.r.t base frame.

(23) $$V_{e}^{b}=\left(\begin{array}{c} v_{e}^{b}\\ \omega_{e}^{e} \end{array}\right)=X_{e}^{b}V_{e}$$

where, $X_{e}^{b}$ is a 6 × 6 spatial motion transformation matrix as a function of end-effector pose w.r.t base frame.

Xeb=[Reb003×3Reb]6×6

The rotational component of the end-effector velocity vector $V_{e}$ is still in the angular velocity form. Whereas, for the input to the robot controller, we need a velocity vector expressed as the rate of change in Euler angles of the end-effector frame. Hence, using the relationship developed in Section 2 Equation (12) for transforming angular velocity to the task space velocity expressed as the rate of change in Euler angles, we have $\omega =B\left(r,p,y\right)*{\left(\begin{array}{ccc} \dot{r} & \dot{p} & \dot{y} \end{array}\right)}^{t}$ comparing with Equation (22) and solving for the rotational velocity $\dot{\chi_{e}}\left(\Gamma \right)$.

(24) $$\dot{\chi_{e}}\left(\Gamma \right)=B{\left(\Gamma \right)}_{m}^{-1}.\omega_{e}^{e}$$

Equation (24) shows the relation between angular velocity and the rate of change in roll, pitch and yaw angles of the end effector, where Γ=(r,p,y), *r* = roll angle, *p* = pitch angle, and *y* = yaw angle of the end-effector frame. Recall from Equation (12):

$$B\left(r,p,y\right)=\left[\begin{array}{ccc} 1 & 0 & \sin p\\ 0 & \cos r & -\cos p\sin r\\ 0 & \sin r & \cos p\cos r \end{array}\right]$$

Now, we can combine the translational vector from base frame velocity vector, and rotational velocity vector expressed as the rate of change in roll, pitch and yaw angles of the end effector, to form a 6 × 1 spatial mix frame velocity vector; Concisely by combining Equations (22) and (24), we can form a single transformation matrix to transform $V_{e}^{e}$ in the desired mix frame velocity $V_{e}^{m}$:

(25) $$V_{e}^{m}=\left(\;\begin{array}{c} v_{e}^{b}\\ {\dot{\chi }}_{e}^{e} \end{array}\right)=X_{e}^{m}V_{e}$$

where:

Xem=[Reb03×303×3B(Γ)−1]6×6

$X_{e}^{m}$ is a 6 × 6 spatial motion transformation matrix, Reb is the 3 × 3 rotation matrix from the end-effector to the base frame, and B(Γ) is the transformation from angular velocity to the rate of change in the end-effector Euler angles. The motion transformation matrix in Equation (25) converts the end-effector frame velocity to an equivalent mix frame velocity $V_{e}^{m}$.

$V_{e}^{m}$ in Equation (25) is the desired mix frame velocity as proposed in this paper, i.e., the end effector’s translation velocity in base frame and rotation velocity expressed as a rate of change in Euler angles of the end-effector frame. $V_{e}^{m}$ is constructed through the Cartesian task space camera velocity $V_{c}$ using Equation (21), needed to converge the IBVS control law. $V_{e}^{m}$ is the minimum norm velocity for a robotic arm end effector due to the use of the pseudo-inverse method in Equation (21). Hence, $V_{e}^{m}$ from Equation (25) can be directly fed to the robot high-level controller to operate the end effector in the Cartesian velocity mode for the desired visual servo control.

Moreover, if a joint control scheme is required while remaining within the framework, one can utilize the developments made in Section 2. Robot-controllers can utilize a mixed frame velocity $V_{e}^{m}$ for visual servoing from Equation (25) and mix frame task Jacobian $J_{m}$ developed in Equation (10), to compute the required joint velocity $\dot{q}$. To implement visual servoing in joint space using mix frame velocity we obtain:

(26) $$\dot{q}=J_{m}^{+}.V_{e}^{m}+\left(I-J_{m}^{+}J_{m}\right)\xi$$

where $J_{m}$ is the mixed frame task Jacobian, $J_{m}^{+}$ is a Moore–Penrose (M-P) pseudoinverse of $J_{m}$**, I** is the identity matrix, and ξ is an arbitrary secondary task vector that can be designed to avoid singularities and joint limits by following the developments in [41,46].

Equation (26) will compute a joint velocity vector $\dot{q}$ with a minimum norm, it implicitly ensures the implementation of required Cartesian velocity in the camera frame and also satisfies the joint limits and singularity avoidance behavior for successfully performing the visual servoing task.

## 4. Experiment & Results

**Figure 7:**
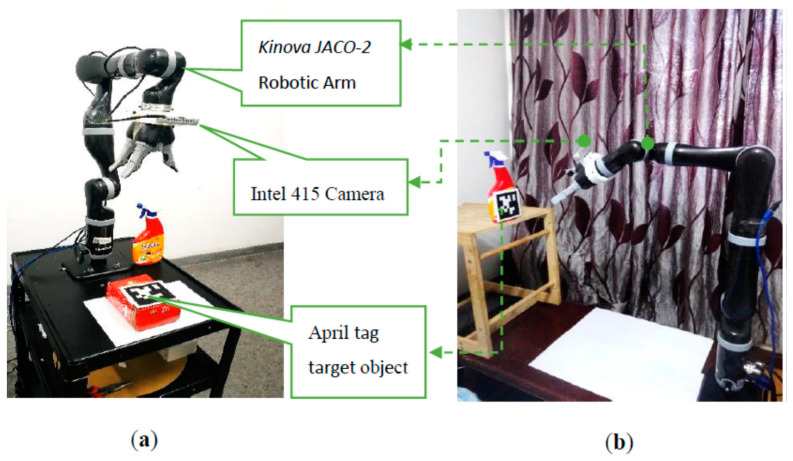
Experimental setup for mix frame IBVS framework using Kinova^®^ 7 DOF robotic arm, Intel D415 camera, April tag target (**a**) lab environment (**b**) home environment.

To evaluate our proposed mix frame visual servo control framework, developed in Section 3, experiments were conducted to achieve an IBVS task with a mixed frame 7 DOF Kinova^®^ *JACO*-2 Assistive robotic arm, an *Intel* RGB-D415 camera in the eye-in-hand configuration and, an April tag [42] target was utilized as shown in the Figure 7, Experimental setup and results are presented in this section.

A *JACO*-2 robotic arm from Kinova^®^ Robotics was selected for the experiments, which is a serial-link stationery, mixed-frame, 7 DOF, spherical wrist ARA. To make the experiment robust to the lighting conditions and evaluate its performance in an unstructured environment, our experiment was conducted under the lab and home environment as shown in Figure 7a,b. Kinova JACO-2 was operated in a mixed frame configuration to perform image-based visual servo control. An *Intel* RGB-D 415 camera was mounted on the robotic arm’s gripper in the eye-in-hand configuration with a constant pose transformation, which can be calculated offline by camera intrinsic and extrinsic calibration by using the Visual Servoing Platform *ViSP*^®^ library [47]. Nevertheless, considering the robustness of IBVS towards camera calibration errors, a coarse estimation will also work.

**Figure 8:**
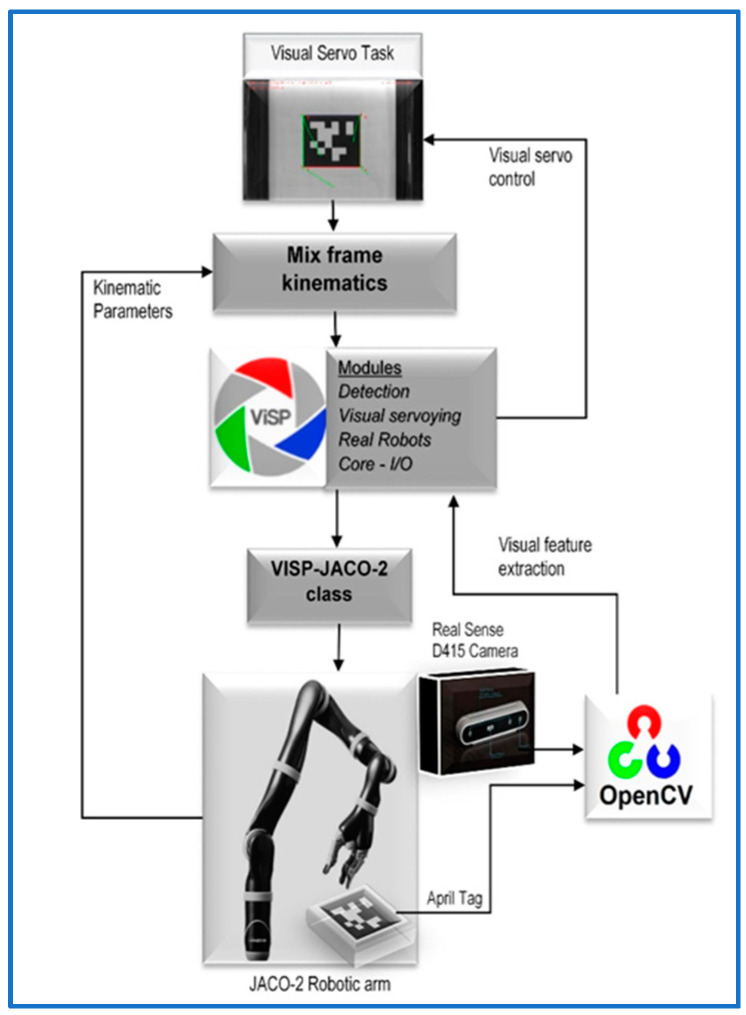
Mixed frame visual servo control experiment.

The program was executed on a desktop PC using an *Intel Core-i5*-8500 CPU with 8GB of RAM, and graphic processing was rendered by a 6 GB *NVidia* graphic card. We noticed that without having a good GPU for the image processing part, the overall program performance becomes sluggish, resulting in longer convergence times, and sometimes visual feature detection failure may occur. The program was developed using C++, *Visual Studio* 2017, *OpenCV*, and the Visual Servoing Platform *ViSP* library [47]. A flowchart of the experiment is shown in Figure 8.

While making this *ViSP* work with the Kinova^®^ *JACO*-2 robotic arm, we developed a new class for *ViSP* library interfacing the Kinova^®^ robotic arm, namely ‘*ViSP-JACO*-2’ with mix frame configuration, it is a much-needed addition in *ViSP* which was not available earlier in the *ViSP* library. It is our major open-source code contribution in the *ViSP* library which is also acknowledged on the *ViSP* developer’s webpage [48] and freely available for use at our GitHub repository [49].

The experiment was divided into sub-parts, A, B, C, and D; where each sub experiment was aimed to investigate a specific aspect of our proposed framework.

**Figure 9:**
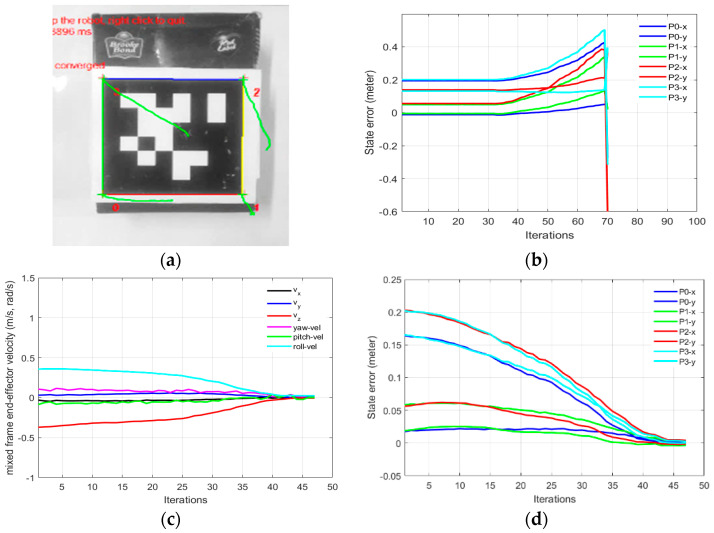
Need for the mix frame visual servo control framework (**a**) feature trajectory for mixed frame velocity (**b**) features error diverging for the end-effector frame (**c**) mixed frame end-effector velocity (**d**) feature error decay for mix frame control.

In Part-A of this experiment, the need for the mixed frame visual servo control framework was established. This experiment describes a conventional IBVS control scheme in the end effector frame, was deployed on a Kinova^®^ *JACO*-2 ARA in Cartesian control mode using Kinova^®^-API high-level controller [33], without the use of the mixed frame control framework. Table 2 shows the initial and the desired feature values of the four corner points of the April tag, used as the visual features for the IBVS as shown in Figure 7. The results are given in Figure 9. While, using the conventional end effector frame configuration for deploying IBVS, the robotic arm rapidly diverges from the task as shown in Figure 9b [see video results-V1], which clearly indicates the need for the development of a mixed frame velocity framework. In Part-A, although the camera velocity Vc, calculated in the camera body frame was converted to the end-effector frame using Equation (22), which is acceptable by industrial robotic arms, it was not compatible with the Kinova^®^ ARA controller that requires velocity in the mixed frame as discussed in Section 3. Therefore, the visual task does not converge and the end effector diverges from the target as seen in Figure 9b, eventually, features leave the camera field of view (video results attached). The visual servo task was failed because the output was Cartesian velocities in the end effector; however, the desired output required by the controller should be in the mixed frame. This was a confusing result for researchers working on the development of visual servo control of assistive mixed frame robots such as Kinova^®^ *JACO-2,* that the robotic arm was not accepting the Cartesian velocities in the end-effector frame.

In part 1-B of this experiment, using our proposed mix frame visual servo controller, the correct mixed frame Cartesian velocities were applied to the controller of the robotic arm, i.e., translation velocities $v_{x,y,z}$ in base frame and $\phi_{y,p,r}$ in the end-effector frame. Results are shown in Figure 9 which demonstrates a successful visual servo control task with sub-millimeter accuracy [see video results-V2]. The visual feature error decay in Figure 9d was fast and smooth. Mixed frame Cartesian velocities were within the maximum velocity limits and converge smoothly as shown in Figure 9c. The initial and desired feature points are shown in Figure 9a where the feature trajectory is almost a straight line. The convergence rate is fast and no overshoots are observed, thanks to the use of an adaptive gain P-D controller which took only 47 iterations to converge to an Euclidean error norm of 0.00005 m.

**Figure 10:**
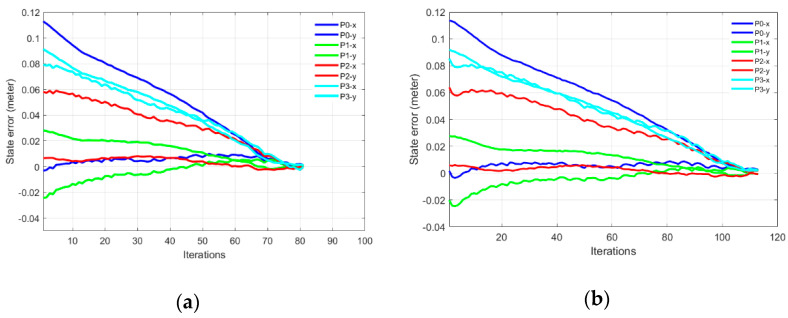
Comparison of an IBVS task between a mixed frame Cartesian velocity controller and a joint velocity controller (**a**) feature error decay for Cartesian velocity (**b**) feature error for joint velocity.

This paper aims to develop an effective IBVS control scheme for the mixed frame ARAs; therefore, we only discuss the relevant design details of a mixed frame adaptive gain PD Cartesian IBVS controller whereas the optimization and a comparison of PID schemes are beyond the scope of this paper. Yet, we briefly present our findings for ARAs comparing the proposed Cartesian mixed frame velocity controller to a joint controller with a constant gain, adaptive proportional gain, and an adaptive proportional derivative controller. For experiment -1, we present a comparison of the mixed frame Cartesian and joint control scheme for ARAs for an IBVS task in Figure 10 and Table 3.

Table 3 summarizes the effect of adaptive gain values on the convergence rate of the task. Starting with a conventional constant gain *P* joint velocity controller using Equation (18) which converged slowly in 446 iterations.

While using an adaptive gain using Equation (20) and a *P* joint controller, the convergence rate was increased with a significant decrease in the number of iterations from 446 to 189, this controller was further improved by introducing adaptive gain *PD* controller and, thus allowing a larger adaptive gain value, resultantly convergence iterations were further reduced to 114.

Increasing gains beyond this point for a joint controller would yield a curve trajectory of the features in the image plane. Taking 114 iterations as a yardstick for comparing our proposed controller with a joint control scheme, a mixed frame Cartesian velocity adaptive gain PD controller using Equation (21) in Equation (25), when deployed on the same task, it converges smoothly in 79 iterations with a substantial increase in the convergence rate by 31% compared with the PD joint controller as shown in Figure 10. This shows the efficiency of our proposed framework compared with the established joint controller.

Generally, while handling end effectors in the Cartesian control mode during conventional IBVS schemes, the manipulator became susceptible to joint limits and kinematic singularities. However, our proposed mix frame IBVS Cartesian control framework for mixed frame ARAs is capable of demonstrating deterrence towards joint limits avoidance and singularity occurrence and is also capable of successfully achieving visual tasks under complex situations.

**Figure 11:**
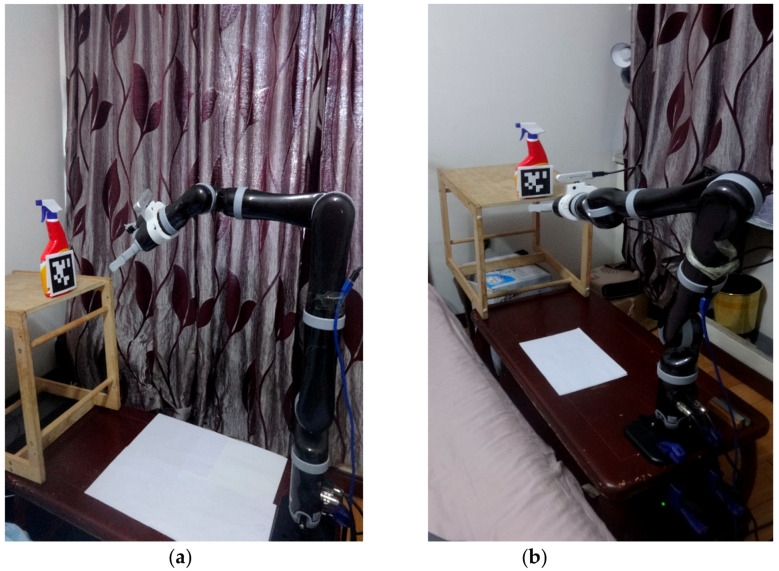

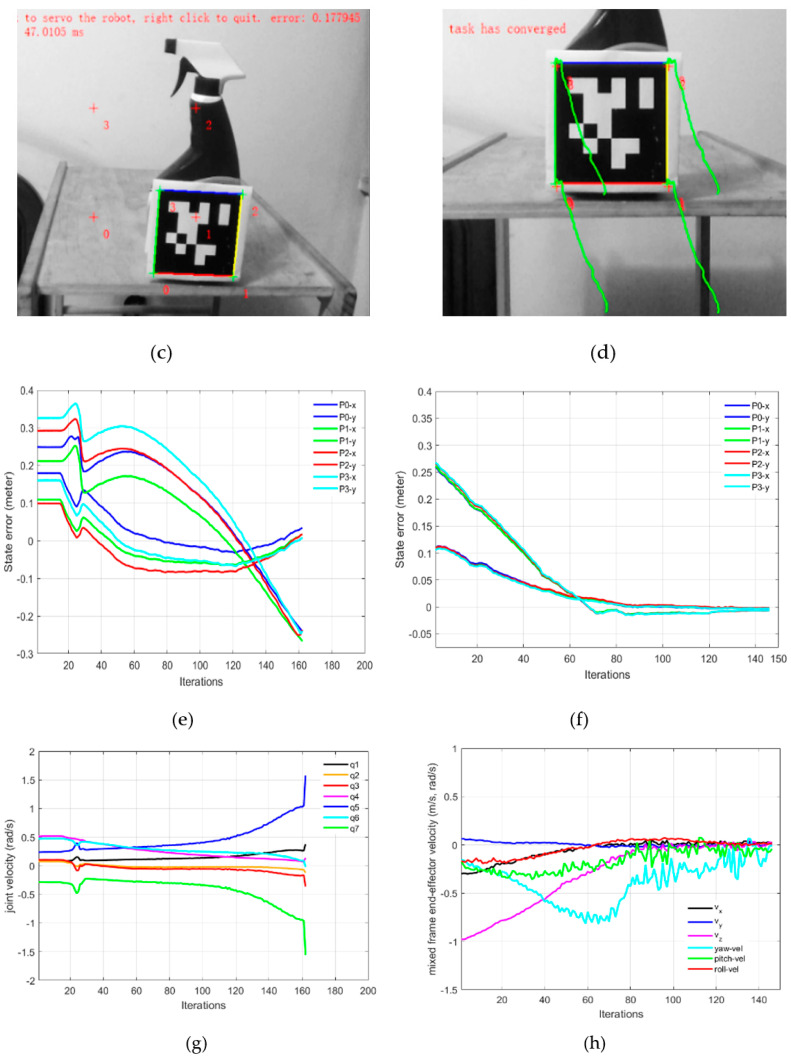
Mix frame visual servo control in extended arm position, (**a**) starting pose, (**b**) end pose, (**c**) initial and desired image features, (**d**) feature trajectory in the image plane for mix frame control, (**e**) feature error in joint control, (**f**) feature error in mix frame control (**g**) joint velocities of joint controlled visual servoing, (**h**) mixed frame end-effector velocity for the proposed control scheme.

This behavior of singularity avoidance is prominent in Part-B of this experiment where the task was defined in the extended arm positions near the boundary of the working envelope of the robotic arm. Figure 11a,b shows the initial and desired pose of the IBVS task, Figure 11c,d shows the initial, the desired feature points and the visual feature trajectory for mix frame Cartesian control. Figure 11e,f shows the feature errors for the mixed frame and joint velocity cases.

The effect of singularity and joint limit occurrence can be observed in Figure 11e–g near the 25th iteration where the arm is struggling to keep its shape in the outstretched position while performing visual servoing [see video results-V3]. Resultantly abrupt changes in joint velocities occurred that leads to the task failure in the conventional joint controller. Whereas the same task was successfully handled by the proposed controller for the arm outstretched position as shown in Figure 11f,h, where smooth decay of feature error can be observed [see video results-V4, V5, V6]. Thanks to the use of task space end-effector mixed frame velocity controller so the joint limits and singularity avoidance behavior were inherently taken care of, by the robotic arm’s controller.

**Figure 12:**
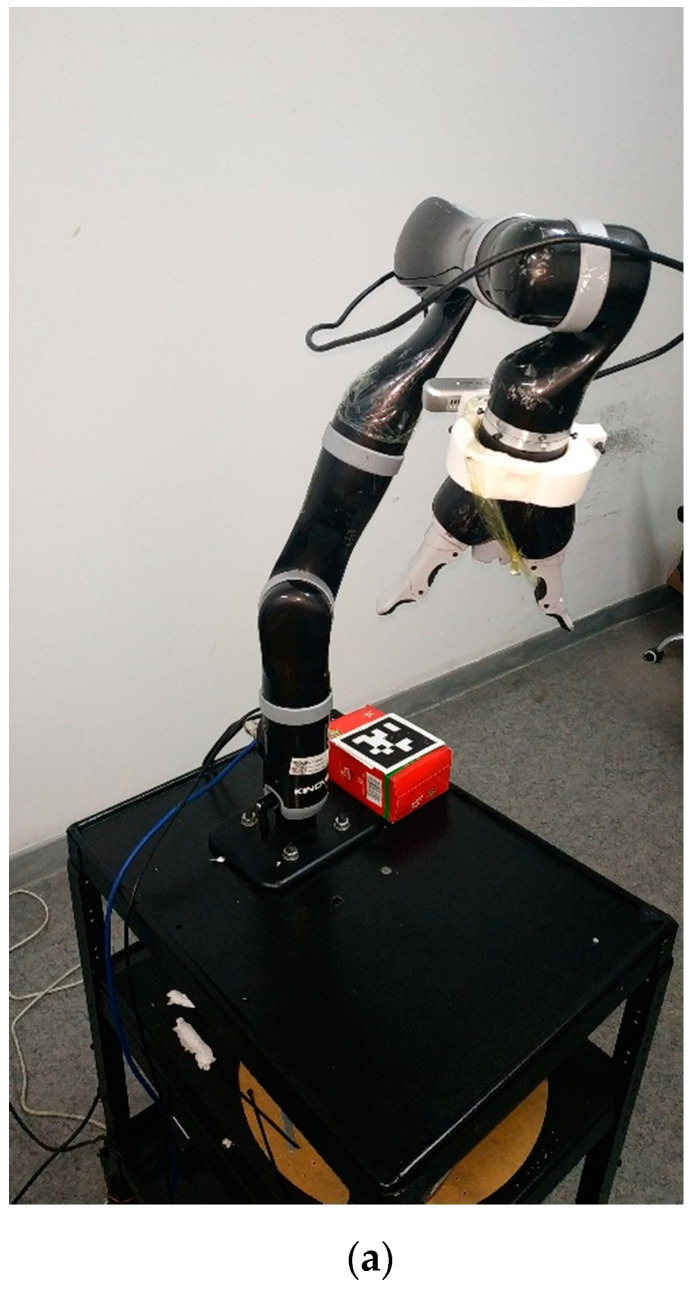

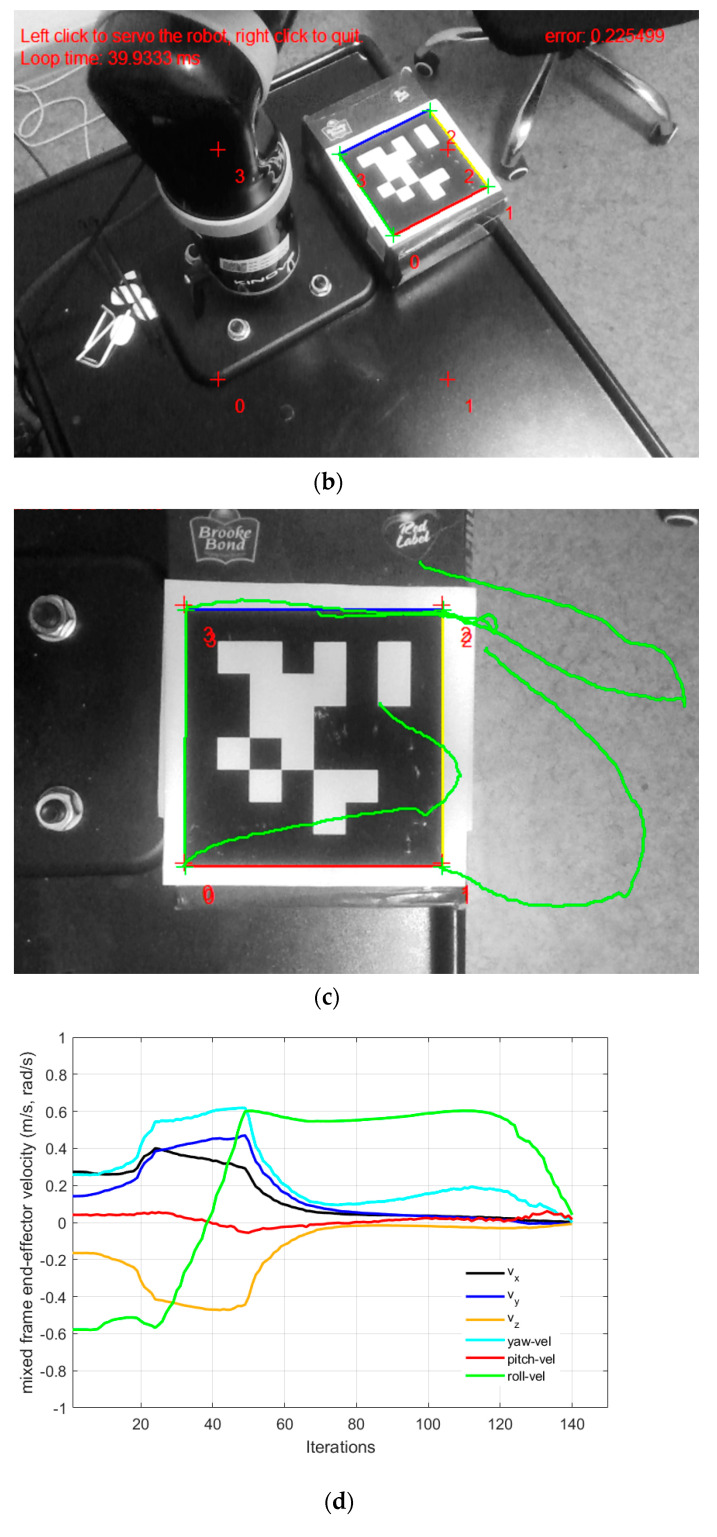

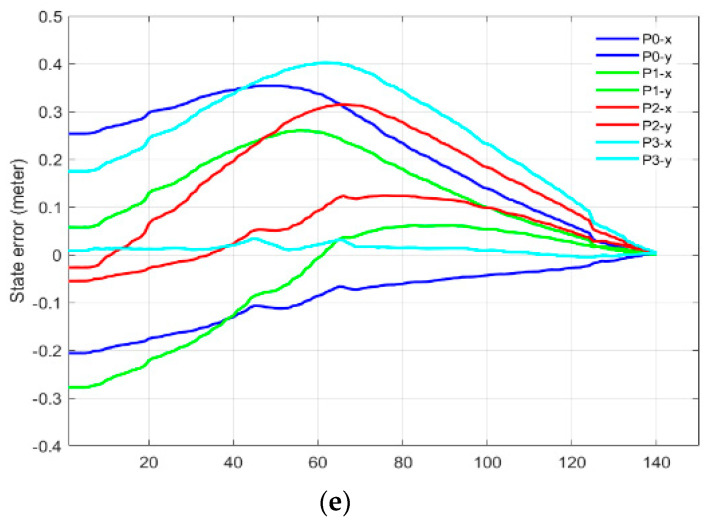
Mix frame visual servo control near the base frame (**a**) starting pose external camera, (**b**) eye-in-hand camera view; initial and desired image features encompassing self-collision, (**c**) feature trajectory in the image plane, (**d**) mix frame end-effector velocity, and (**e**) visual feature error decay in the mixed-frame.

In part-C of this experiment, the IBVS task was completed successfully near the base frame. Starting from an initial pose where the self-collision of the arm is expected during the visual servoing as shown in Figure 12a,b. Nevertheless, our proposed mix frame visual servo controller in the Cartesian control mode successfully achieved this IBVS task without stopping or colliding with the robotic arm, rather it glides over the safety zone defined near the base of the robotic arm as seen in feature trajectory Figure 12c,d [see video results-V7]. Please note points 0 and 3 cannot be derived in a straight line towards the target features, if they do, it would collide with the end effector in the base of the robotic arm, therefore the controller forced the image points to travel away from the base frame avoiding to come near the body of the robotic arm, which forced the image points to take a curved route to their desired feature position, as can be observed in Figure 12c. The results in Figure 12 show the visual features trajectory, feature error decay, and the mix frame velocity for this part of the experiment.

As shown in part-B and part C of this experiment in Figure 11 and Figure 12, while operating the arm in the outstretched condition and near the base frame, where the robotic arm was suspected for singularities, joint limits and self-collision. Our proposed mix frame IBVS controller was capable of demonstrating deterrence towards joint limits, singularities, and self-collision problems and successfully achieved visual tasks under complex situations thanks to the use of Kinova^®^ ARAs low-level controller’s inherent ability [30,35] to avoid these constraints when operated in the mixed frame Cartesian velocity control mode. We achieved this behavior without the use of an external singularity avoidance and joint limits algorithm, which needs to be implemented otherwise as a secondary task, if used in conjunction with a joint controller [37,41]. For generic robotic arms, these features can be separately included in the framework following the developments made in [46,50].

After repetitive trials, none of the experiments had failed due to joint limits, kinematic singularity, or self-collision occurrence in the manipulator, which shows the robustness of our proposed framework.

**Figure 13:**
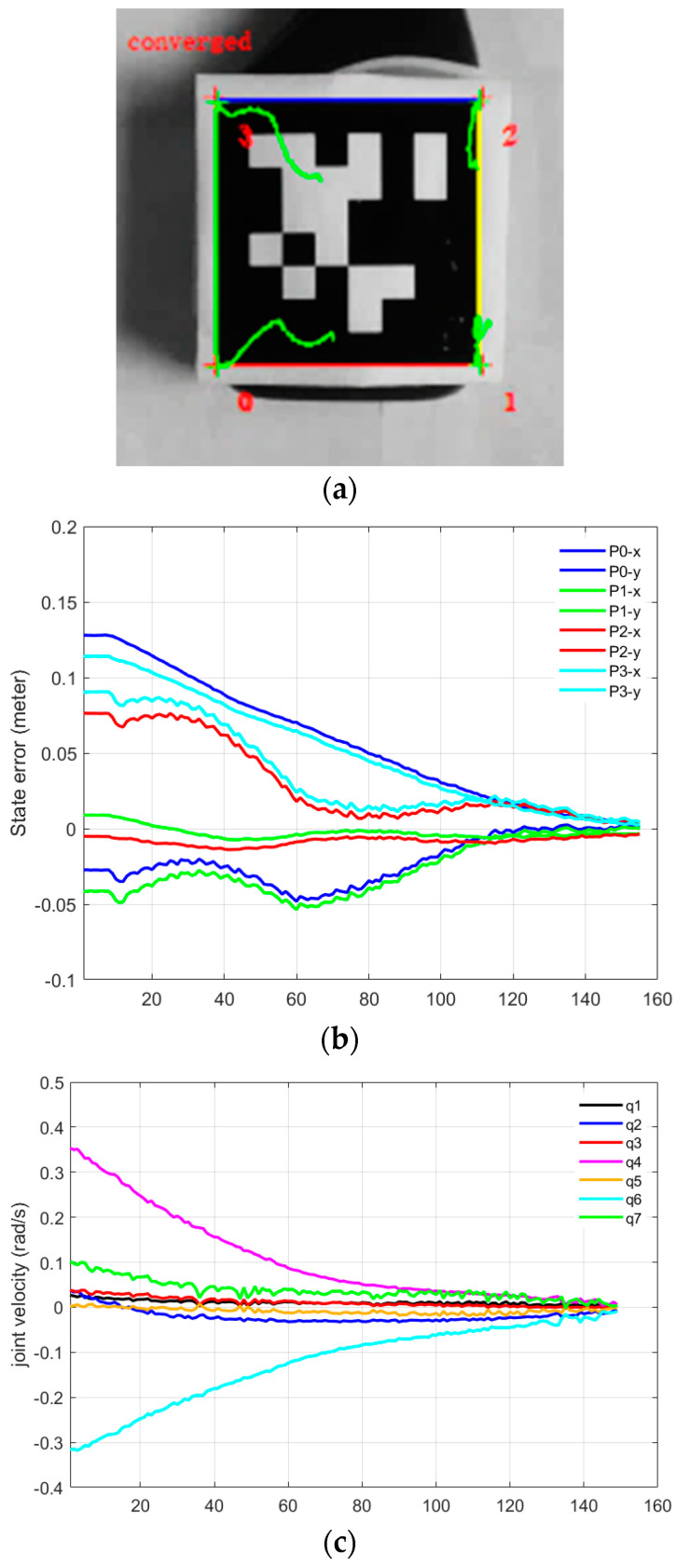
Visual servoing in the joint space using mix frame velocity and mix frame Jacobian (**a**) feature trajectory in the image plane, (**b**) visual feature error decay, (**c**) joint velocities.

In Part-D of this experiment, we validated joint velocity control through our proposed framework, in which our newly developed mix frame Jacobian from Section 2, and mixed frame end-effector velocity from Section 3, were utilized using Equation (26) to implement a joint velocity controller to achieve an IBVS task. Figure 13a–c shows the results of the experiment including visual feature trajectories, error decay, and the manipulator’s joint velocities.

An important observation to note in Part-D is that the task took 154 iterations for convergence with a wavy image point trajectory [see video results-V8]; however, with a stabilized decreasing joint velocity as shown in Figure 13c. This behavior is apprehensible, considering that, even if the robotic arm is 6 DoF or it may be redundant, generally it is not identical to compute first the *V_m_* using Equation (25) and then calculating the corresponding joint velocities $\dot{q}$ using the mixed frame Jacobian in Equation (26), or on the other hand, to directly compute $\dot{q}$ for visual servo control using Equation (18). Nevertheless, both techniques are correct, yet the two control schemes are different and will produce two different joint velocities and image point trajectories. Actually, it may happen that the manipulator Jacobian may be singular whereas the feature Jacobian is not (that occurs if k < n). Moreover, the use of pseudo-inverse in Equation (21) ensures that camera velocities $V_{c}$ are minimal while in this case for Equation (26), joint velocities $\dot{q}$ are minimal. Hence, the choice of the state space variable is vital.

## 5. Conclusions

In this paper, a novel mixed frame visual servo control framework was developed using task space kinematics of ARAs in the Cartesian space to induce autonomous behavior in the assistive robotic arms. The need for the proposed framework has emerged as currently available visual servo controllers were incompatible with ARAs embodied mixed frame kinematics, which limits the use of mainstream visual servo controllers with ARAs. In this framework, a mixed frame adaptive proportional derivative IBVS control was developed for ARAs where camera velocities were transformed into the mixed frame end-effector velocities expressing translation velocity in the base frame, and the orientation velocity was expressed as the rate of change in the Euler angle of the end effector. Using the proposed mixed framework, ARAs can efficiently perform visual servoing in the Cartesian velocity control mode using its embedded mixed frame kinematics, thereby preserving its core functionality and safety features.

We presented the results for ARAs performing complex IBVS tasks precisely, for objects positioned near the base of the robotic arm and at the end of the work envelope. These tasks were not previously achievable using conventional visual servoing controllers. The proposed framework is efficient, robust to common manipulation errors, and expandable to other ARAs that can operate under Cartesian velocity control. Thanks to the use of the mixed frame Cartesian control mode, modern robotic arms such as Kinova^®^ *JACO* and MICO series can inherently avoid joint limits, singularities, and self-collision.

The scientific contributions of this paper are:

- Identifying the correct kinematic structure of ARAs and developing the mixed frame task space kinematics of ARAs while highlighting the benefits of the mixed frame configuration;
- Introducing an innovative concept of a “mixed frame task velocity” and the “mixed frame Jacobian” for directly solving the inverse kinematics of mixed frame robotic arms;
- Development of a “mixed frame visual servo control framework” for visual servo control of ARAs, while safeguarding their embodied mixed frame kinematics and core safety features;
- Experimental validation of the proposed framework on a 7 DoF Kinova^®^ *JACO*-2 assistive robotic arm using mixed frame Cartesian velocity along with adaptive *PD* IBVS controller, that can achieve sub-millimeter accuracy for an IBVS task with a 31% significant increase in the convergence rate as compared to the conventional IBVS controllers.

Nevertheless, we also noted some limitations of this framework: it is designed for eye-in-hand configuration, yet it can be extended to the eye-to-hand case with related changes in Section 3. Besides some features of this framework are more beneficial when used in conjunction with modern ARAs having built-in safety features such as Kinova^®^ robotic arms, although such features can be added separately as explained in Section 4.

This research work will bridge the gap between the current advances in robotics research and assistive robotics technology while safeguarding the core safety features. In the future, the mixed frame visual servo control framework will be expanded to include markerless visual servoing in the unstructured environments. Another interesting scenario would be to investigate the use of a mixed frame configuration for an industrial robotic arm.

## Figures and Tables

**Table 1:** *D-H* Parameters of a Kinova^®^ *JACO*-2 7-*DoF* Assistive robotic arm.

**i**	$\alpha_{i-1}$	$a_{i-1}$	$d_{i}$	$\theta_{i}$
1	π/2	0	−D1	q1
2	π/2	0	0	q2
3	π/2	0	−(D1 + D3)	q3
4	π/2	0	−e2	q4
5	π/2	0	−(D4 + D5)	q5
6	π/2	0	0	q6
7	π	0	−(D6 + D7)	q7

**Table 2:** Image points feature list for the IBVS Experiment-1.

Image Point	X_0_	Y_0_	X_1_	Y_1_	X_2_	Y_2_	X_3_	Y_3_	Z (Depth)
Initial **s**	0.1019	0.0713	0.0802	0.0571	0.0659	−0.125	−0.1162	0.1107	0.5247
Desired s*	−0.1666	0.1666	0.1666	0.1666	0.1666	−0.1666	−0.1666	−0.1666	0.2888

**Table 3:** Effects of adaptive gain on the convergence rates of IBVS controller.

**IBVS** **Controller**	Gain Values			Convergence (Iteration)
	*λ*	$\lambda_{adp}$	$k_{d}\;$ *(s)*	
Constant gain P -Joint velocity.	1.2	-	-	446
Adp. Gain P -Joint velocity.	_	$\lambda_{0}$ = 2.0 $\lambda_{\infty }$= 0.2 $\lambda_{0}^{\prime }$ = 30°	_	189
Adp. Gain PD -Joint velocity.	_	$\lambda_{0}$ = 2.8 * $\lambda_{\infty }$= 0.3 $\lambda_{0}^{\prime }$ = 30°	0.25	114
Adp. Gain PD -Cartesian velocity. (Proposed)	_	$\lambda_{0}$ = 4.5 $\lambda_{\infty }$= 0.5 $\lambda_{0}^{\prime }$ = 30°	0.55	79

P: proportional; $k_{d}$ = derivative gain; Adp: Adaptive gain; PD: Proportional Derivative. * Beyond this value feature trajectory becomes curved in our case.

## Data Availability

No public data set available.

## Supplementary Materials

The following videos from the Mixed frame visual servo control experiment are available online at https://www.mdpi.com/article/10.3390/s22020642/s1, Video Supplementary; (1). IBVS of ARAs using conventional end-effector frame. (2). IBVS of ARAs using the proposed mixed frame adaptive PD Cartesian IBVS velocity controller. (3). IBVS of ARAs using a conventional joint controller in the arm outstretched position. (4). IBVS of ARAs using proposed mixed frame configuration in the arm outstretched position; (5). IBVS of ARAs using proposed mixed frame configuration with Different starting poses; (6). IBVS of ARAs using proposed controller with Higher adaptive gain values (fast, stable but curved image feature trajectory). (7). IBVS of ARAs using proposed mixed frame configuration near the base frame avoiding self-collision. (8). IBVS of ARAs in the joint space using proposed mixed framework and the mixed frame Jacobian.

## Nomenclature

$V_{e}^{m}$	End-effector twist vector in the mixed frame
$V_{e}^{b}$	End-effector twist vector in the base frame
q	Robotic arm joint angles
$\dot{q}$	Robot’s Joint velocity vector
ω	Angular velocity
r,p,y	Roll, pitch, yaw angles of the end-effector frame
$\chi_{conv}$	Pose in a conventional task space
$\chi_{mix}$	Pose in the mixed frame configuration
$\dot{\chi }\;$	Task space velocity of the end effector
$\dot{\phi_{e}}\left(\Gamma \right)$	Rotational velocity of the end effector as the rate of change in Euler angles
$v_{e}^{b}$	End-effector translation velocity in the base frame
$V_{e}^{e}$	End-effector velocity vector in the end-effector frame
$V_{c}^{c}$	Camera twist vector in the camera frame
$p_{x,y,z}^{b}$	Position of the end-effector w.r.t the base frame
$J_{mix},J_{m}$	Mixed frame Jacobian matrix
J(Γ)	Analytical Jacobian transformation matrix
$J_{b}$	Jacobian matrix in the base frame
λ	Constant proportional gain
$\lambda_{adp\;}$	Adaptive gain
$k_{d\;}$	Derivative gain
$\dot{e}\;$	Rate of change in feature error
X	Motion transformation matrix
B	Analytical transformation matrix
Rce	Rotation matrix from the camera to the end-effector frame
Reb	Rotation matrix from the end effector to the base frame
Rbe	Rotation matrix from the base to the end-effector frame
